# Prognostic and Predictive Biomarkers in Gliomas

**DOI:** 10.3390/ijms221910373

**Published:** 2021-09-26

**Authors:** Paulina Śledzińska, Marek G. Bebyn, Jacek Furtak, Janusz Kowalewski, Marzena A. Lewandowska

**Affiliations:** 1 Department of Thoracic Surgery and Tumors, Ludwik Rydygier Collegium Medicum in Bydgoszcz, Nicolaus Copernicus University, 85-067 Torun, Poland; paula.sledzinska@gmail.com (P.Ś.); kowalewskij@co.bydgoszcz.pl (J.K.); 2 The F. Lukaszczyk Oncology Center, Molecular Oncology and Genetics Department, Innovative Medical Forum, 85-796 Bydgoszcz, Poland; marek.bebyn@gmail.com; 3 Faculty of Medicine, Medical University of Gdańsk, 80-210 Gdańsk, Poland; 4 Department of Neurosurgery, 10th Military Research Hospital and Polyclinic, 85-681 Bydgoszcz, Poland; jacek.furtak2019@gmail.com; 5 Franciszek Lukaszczyk Oncology Center, Department of Neurooncology and Radiosurgery, 85-796 Bydgoszcz, Poland

**Keywords:** biomarker, brain neoplasms, gliomas, liquid biopsy, predictive value, prognosis, WHO CNS5

## Abstract

Gliomas are the most common central nervous system tumors. New technologies, including genetic research and advanced statistical methods, revolutionize the therapeutic approach to the patient and reveal new points of treatment options. Moreover, the 2021 World Health Organization Classification of Tumors of the Central Nervous System has fundamentally changed the classification of gliomas and incorporated many molecular biomarkers. Given the rapid progress in neuro-oncology, here we compile the latest research on prognostic and predictive biomarkers in gliomas. In adult patients, *IDH* mutations are positive prognostic markers and have the greatest prognostic significance. However, *CDKN2A* deletion, in IDH-mutant astrocytomas, is a marker of the highest malignancy grade. Moreover, the presence of *TERT* promoter mutations, *EGFR* alterations, or a combination of chromosome 7 gain and 10 loss upgrade IDH-wildtype astrocytoma to glioblastoma. In pediatric patients, *H3F3A* alterations are the most important markers which predict the worse outcome. *MGMT* promoter methylation has the greatest clinical significance in predicting responses to temozolomide (TMZ). Conversely, mismatch repair defects cause hypermutation phenotype predicting poor response to TMZ. Finally, we discussed liquid biopsies, which are promising diagnostic, prognostic, and predictive techniques, but further work is needed to implement these novel technologies in clinical practice.

## 1. Introduction

Gliomas, broadly categorized by their cell of origin, are the most common central nervous system (CNS) tumors [1]. Gliomas account for ~30% of all primary brain tumors, 80% of all malignant ones, and the vast majority of deaths caused by primary brain tumors [2]. The incidence of gliomas in adults varies from 1.9 to 9.6 per 100,000 depending on age, sex, ethnicity, and geographic location [3,4]. The median age at diagnosis varies by histological subtype, with pilocytic astrocytomas occurring more frequently in children and adolescents, low-grade oligodendrogliomas peaking in the third and fourth decades, and glioblastomas mainly presenting in patients over 50 years of age [5,6]. Although most gliomas occur in the four lobes of the brain [frontal (23.6%), temporal (17.4%), parietal (10.6%), and occipital (2.8%)], a small proportion can appear in the brain stem, cerebellum, and spinal cord [3]. Survival outcomes are largely dependent on grade, with CNS World Health Organization (WHO) grade 1 having the best relative survival [7,8], and CNS WHO grade 4 having the worst overall survival (OS) rate, with just 6.8% of patients living for five years after diagnosis [7]. 

The initial management of gliomas usually consists of maximally safe surgical resection, which in addition to reducing tumor volume allows for tissue acquisition for an accurate histological diagnosis and tumor genotyping [9]. This is often followed by radiotherapy (RT) and temozolomide (TMZ) chemotherapy [10]. The emergence of tumor-treating fields (TTFields)—low-intensity alternating electric fields that disrupt mitosis at the metaphase to anaphase transition—has also recently emerged as a promising modality to improve standard of care [11,12]. Furthermore, TTFields alter cellular membranes, rendering cells more permeable to chemotherapeutics [13], so when added to standard of care in randomized phase III clinical studies, TTFields increase life expectancy by four months with minimal side-effects [14].

Prognostic and predictive markers play an important role in clinical practice for the assessment of prognosis and the selection of appropriate therapy. This is especially important in gliomas due to the possible occurrence of so-called pseudoprogression in MRI [15]. Pseudoprogression is chemo- and radiation-induced brain tissue reaction that resembles true tumor progression in 30% of patients receiving standard of care for glioblastoma (GBM), which could be distinguished based on biomarker analysis [16]. Moreover, given advances in sequencing technologies and resulting knowledge about genetic changes occurring in tumors, new biomarkers are expected to significantly improve patient management. Due to the rapid progress in neuro-oncology, here we compile the latest research on prognostic and predictive biomarkers in gliomas.

## 2. The 2021 WHO Classification of Tumors of the Central Nervous System 

The fifth edition of the WHO Classification of Tumors of the Central Nervous System (WHO CNS5) is the current international standard for glioma nomenclature and diagnosis. Traditionally, CNS tumor grading has been based exclusively on histological features, but certain biomarkers can now provide powerful prognostic information. Therefore, molecular parameters have been added to more precisely grade gliomas and for further estimating prognosis within multiple tumor types. In 2016, the WHO CNS classification for the first time used molecular markers to classify gliomas, and in 2021, placed even more emphasis on them [1,17]. Numerous molecular alterations with clinicopathologic usefulness are included in WHO CNS5 [1]. In this classification system, the primary genetic markers for gliomas are *IDH* mutation status, codeletion of chromosomal arms 1p and 19q (1p/19q codeletion), *H3F3A* alterations, nuclear alpha-thalassemia/mental retardation X-linked syndrome (*ATRX*) gene mutations, O^6^-methylguanine-DNA methyltransferase (*MGMT*) promoter methylation status, loss of cyclin-dependent kinase inhibitor 2A (*CDKN2A*), epidermal growth factor receptor (*EGFR*) amplification, combined gain of chromosome 7 and loss of chromosome 10 (7+/10−), and telomerase reverse transcriptase (*TERT*) promoter mutations [18]. The great number of biomarkers not only indicates a paradigm change in classification, but it also has implications for how patients with these malignancies are managed clinically.

Moreover, several major changes have been implemented in the novel classification. WHO CNS5 has developed a new method to classify gliomas and grouped them into six different families (see Table 1). Importantly, WHO CNS5 distinguishes between diffuse gliomas that predominantly affect adults (referred to as “adult-type”) and those that primarily affect children (referred to as “pediatric-type”). Neoplasms are graded within types using Arabic numerals rather than across different tumor types using Roman numerals [19]. The terms diffuse or anaplastic are no longer used to describe the grade of malignancy. This simplifies the classification, for example astrocytoma, IDH-mutant covers grades 2–4 and eliminates the terms “glioblastoma, IDH-mutant”, “diffuse astrocytoma”, “anaplastic astrocytoma”. Moreover, in the context of a pediatric-type tumor, the term “glioblastoma” is no longer used. Furthermore, previously, certain tumor names included anatomic site modifiers, while others did not, despite the fact that they occurred in specified areas. Names have therefore been simplified as much as possible, and only location, age, or genetic modifiers with clinical utility have been used [1].

## 3. IDH1 and IDH2

**Figure 1:**
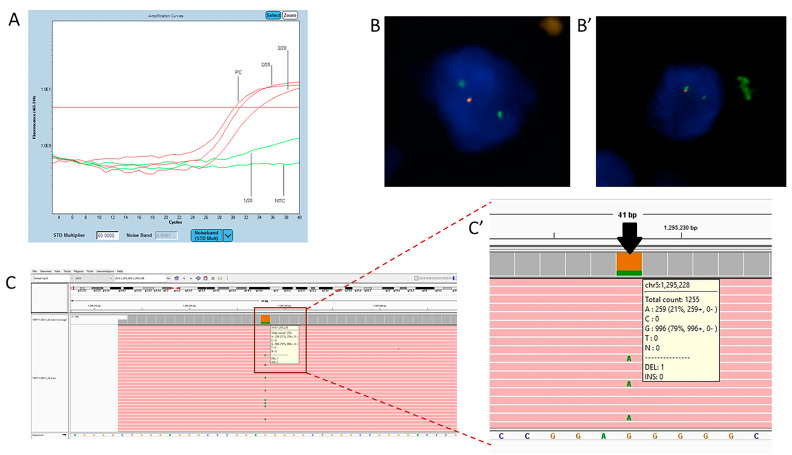
Application of fluorescence in situ hybridization (FISH), next-generation sequencing (NGS), and quantitative polymerase chain reaction (qPCR) in the genetic diagnosis of glioma. (**A**) Representative screenshot of an isocitrate dehydrogenase 1 (*IDH1*) mutation. Red amplification curves represent positive signals for the mutation in exon 4, codon 132 of the IDH1 gene detected in positive control (PC) and two patients’ samples (2/20 and 3/20). The test does not distinguish between *IDH1* changes: R132H (c.395G>A), R132C (c.394C>T), R132S (c.394C>A), R132G (c.394C>G), R132L (c.395G>T), R132P (c.395G>C). Sample 1/20 negative for the presence of mutations in *IDH1* codons 132 and 100 and the IDH2 gene at codons 140 and 172 (IDH-RT38, Entrogen). (**B**) Applications of FISH in genetic diagnostics in glioma on biological material collected by stereotactic biopsy. Deletion of 1p32 (cell on the left). (**B’**) Deletion of 19q13 (cell on the right) (Abbott, Molecular). (**C**) Representative screenshot of a telomerase reverse transcriptase (*TERT*) variant using Integrative Genomics Viewer (IGV). Aligned NGS data produced with Entrogen’s Targeted Hotspot Panel 16 kit. (**C’**) Close-up of *TERT* promoter variant NC_00005.9: g.1295228G>A; (commonly called C228T mutation) Reported as pathogenic.

Recurrent mutations in the active site of *IDH1,* occurring in 12% of malignant gliomas, were first reported in 2008 [20]. Although *IDH1* and *IDH2* are highly similar and catalyze identical reactions, their expression differs in different cancers and their subtypes. *IDH1* mutations predominate in gliomas [21,22] and are nearly all caused by a single amino acid substitution at codon 132 (Figure 1A) [23]. Cancer-associated *IDH1* mutations produce *R*(−)-2-hydroxyglutarate (2HG) instead of α-ketoglutarate [24], the latter altering cancer metabolism and creating oxidative stress [25]. Alpha-ketoglutarate levels influence the hypoxia-inducible factor subunit HIF-1α, a transcription factor that promotes tumor growth when oxygen levels are low [26] and also inhibit histone demethylation, which is essential for the terminal differentiation of lineage-specific progenitor cells [27]. NADPH production is impaired in gliomas with *IDH1* mutations, which may sensitize the tumors to radiation and chemotherapy, explaining why patients with *IDH* mutant neoplasms live longer [28]. Moreover, *IDH1* mutations occurred in a substantial proportion of patients, who were on average 17 years younger than patients who did not have this abnormality [20,28,29]. 

*IDH* mutations play a crucial role in glioma classification. Especially, in adult-type diffuse gliomas where all types require *IDH* assessment (see Table 1). Currently, to classify a tumor as oligodendroglioma both the IDH-mutation and 1p/19q codeletion should be identified [1]. Moreover, in IDH-wildtype diffuse astrocytic gliomas the presence of one or more of three genetic parameters (*EGFR* gene amplification, *TERT* promoter mutation, 7+/10−) appears to be adequate to assign the highest CNS WHO grade and classify them as glioblastoma-wildtype. Such an approach avoids confusion and makes it easier to include these patients in clinical trials [30]. Besides, the Cancer Genome Atlas (TCGA) research network indicated that *IDH*, 1p/19q, and *TP53* status captured lower-grade glioma subtypes more precisely than histological classification [31]. 

### Positive Prognostic Factors

Mutations in *IDH1* or *IDH2* are positive prognostic factors (see Table 1). In comparison to IDH-wildtype gliomas, patients with IDH-mutant gliomas have a much better prognosis [16,26,27,32,33,34,35,36,37]. Large meta-analyses have shown that *IDH* mutations are associated with longer overall survival (OS) and progression-free survival (PFS) [38,39], regardless of grade [40]. The most favorable clinical outcomes were observed in lower-grade gliomas with *IDH* mutations and 1p/19q codeletion [31]. In malignant gliomas, the combination of *IDH1* mutations and *MGMT* methylation status is more predictive of survival than either *IDH1* or *MGMT* alone [40]. The multigenic mechanism behind its prognostic value is very much in line with the current observations done in the mechanistic pan-cancer studies. The activity of specific metabolic modules shows a stronger association with survival than any of its gene components alone [41].

In adult patients, *IDH* mutation has the greatest prognostic significance and clinical utility. Therefore, it should be assessed first and foremost.

## 4. H3F3A

H3.3 is a universal, replication-independent histone mostly found at transcription start sites and in telomeric regions, and it is associated with active and open chromatin [42,43]. The H3.3 histone is encoded by two different genes, *H3F3A* and *H3F3B* [44]. *H3F3A* alterations define an epigenetic subgroup of high-grade gliomas (HGG) with distinct clinical features and a global methylation pattern [30,44,45,46,47]. *H3F3A* mutations affect two amino acids, K27 and G34 of H3.3, in one-third of pediatric malignant gliomas. There appear to be strong links between *H3F3A* alterations and the age of onset of HGG, with the *H3F3A* K27 mutation occurring in children and *H3F3A* G34 in adolescents and young adults [44]. Moreover, H3.3 K27M mutations mainly occur in thalamic malignant neoplasms, whereas H3.3 G34R or G34V mutations tend to occur in cerebral hemisphere tumors [44,46]. As a result, WHO CNS5 classified tumors with alterations in H3F3A to the pediatric-type diffuse high grade gliomas family (see Table 1) [1]. Diffuse midline glioma, H3 K27-mutant, was previously described in the 2016 WHO classification. However, the nomenclature has been modified as H3 K27-alterted to reflect the fact that other molecular alterations can explain this type in addition to the previously known H3 K27 mutation [1,17].

### Negative Prognostic Factor

Brain tumors are the leading cause of cancer-related death in children [47]. Most childhood brain tumors are CNS WHO grade 1, but the *H3F3A* alterations are associated with significantly worse outcomes (see Table 1) [45]. The H3 K27-altered diffuse midline glioma is an aggressive tumor corresponding to CNS WHO grade 4. Similarly, detection of an H3 G34 mutation in a diffuse glioma, irrespective of histological grade, indicates high-grade biology [30]. The K27M mutation represents a particularly unfavorable group, with a three-year OS of only 5% [48]. Overall, in pediatric patients, the determination of protein K27 and G34 defects is of the greatest clinical importance.

## 5. ATRX

*ATRX* is a key component of a multiprotein complex that also contains death-associated protein 6 (DAXX). *ATRX* regulates chromatin remodeling, nucleosome assembly, telomere maintenance, and histone H3.3 deposition in transcriptionally silent genomic regions [49]. ATRX protein loss and *ATRX* gene mutations are hallmarks of genomic instability and ALT-immortalized cell lines [50,51]. *ATRX* is frequently mutated in astrocytomas, IDH-mutant (about 60–70%) [52]. Diffuse astrocytic tumors harboring *IDH* mutations can be diagnosed as astrocytoma, IDH-mutant if there is loss of ATRX nuclear expression and/or diffuse p53 immunopositivity without the need for 1p/19q testing [30]. Moreover, loss of nuclear ATRX expression is one of the criteria to diagnose high-grade astrocytoma with piloid features [19]. 

### Prognostic Factor

Pekmezci et al. reported that *ATRX* alterations were not associated with survival in astrocytomas IDH-mutant (see Table 2). On the other hand, in glioblastomas IDH-wildtype, *ATRX* alterations were associated with favorable outcomes [53]. Therefore, further research is required to assess its prognostic value. 

## 6. TERT

The telomerase reverse transcriptase gene encodes the catalytic reverse transcriptase subunit of telomerase that maintains telomere length. Somatic *TERT* mutations involving regulatory regions in addition to coding sequences may represent important driver events in cancer [76]. Mutations in the coding region of *TERT* are uncommon, but mutations in the promoter region have been detected in a high percentage of human melanomas and metastatic cancers [77]. Two well-established *TERT* promoter mutations result from cytidine-to-thymidine transitions in a dipyrimidine motif (C228T and C250T) (Figure 1C). The mutations create an alternative binding motif for ETS transcription factors and ternary complex factors (TCFs) near the transcription start site, resulting in up to a two-fold increase in transcription [76]. *TERT* promoter mutations reactivate telomerase, allowing for indefinite telomere maintenance and enabling cellular immortalization [78,79]. The C228T mutation accounts for 75% and C250T for 25% of all *TERT* promoter mutations [80] (Figure 1C). Approximately 70% of all adult primary glioblastomas harbor *TERT* promoter mutations [81,82]. Moreover, *TERT* promoter mutations were observed in almost all gliomas with concurrent total 1p/19q loss and *IDH1*/2 mutations (98%) [79].

### Negative Prognostic Factor 

Various studies have reported that telomerase activation or increased *TERT* expression are associated with shorter survival in gliomas [83,84,85]. In their meta-analysis, Vuong et al. reported that *TERT* promoter mutations are significantly associated with worse OS and PFS (see Table 1). Moreover, they noted that the prognostic significance of *TERT* mutations depends upon *IDH* status and tumor histology [61]. 

*TERT* promoter mutation is a negative prognostic factor, but mainly in IDH-wildtype gliomas. Pekmezci et al. reported that in astrocytomas IDH-wildtype, the *TERT*-wildtype group had a significantly better OS than the *TERT*-mutated group. In contrast, *TERT*-mutated tumors were not associated with survival in astrocytomas IDH-mutant. Moreover, in oligodendrogliomas, patients in the *TERT*-wildtype group had significantly worse OS than those in the *TERT*-mutated group [53]. Finally, *TERT* promoter mutation is one of the three genetic parameters that WHO CNS5 uses to upgrade astrocytoma, IDH-wildtype to glioblastoma, IDH-wildtype [1]. As a result, additional research is necessary to determine its multigenic relationship.

## 7. CDKN2A

Cyclin-dependent kinase inhibitor 2A (*CDKN2A*) is located on the short arm of chromosome 9 (p21.3) [86]. In several tumor subtypes, homozygous deletion of *CDKN2A* is associated with increased carcinogenesis and a poor prognosis [65]. The presence of a homozygous deletion of cyclin-dependent kinase inhibitor 2A/B (CDKN2A/B) is now playing an important role in glioma classification as a negative prognostic biomarker.

### Negative Prognostic Factor 

Many studies have reported that *CDKN2A* deletion is associated with significantly shorter PFS and OS in both lower-grade glioma (LGG) and HGG (see Table 1) [48,65,86,87,88,89,90,91,92]. The *CDKN2A* homozygous deletion is a significant prognostic factor in IDH-mutant glioma patients across multiple histologic WHO grades [65]. Ghasimi et al. reported that *CDKN2A/B* risk genotypes are also related to glioblastoma IDH-wildtype [93]. Allelic loss of *9p21.3*, which contains *CDKN2A*, is a prognostic factor in 1p/19q-codeleted grade 3 gliomas. Allelic loss of 9p21.3, detected in 41.7% of tumors, was associated with shorter PFS and OS in univariate analysis [94]. Based on the robust literature and the cIMPACT-NOW update 5 recommendations, astrocytoma, IDH-mutant that harbors homozygous CDKN2A/B deletion is graded as CNS WHO grade 4. In other words, the presence of homozygous CDKN2A/B deletion is a marker of the highest malignancy grade in the group of diffuse, IDH-mutant astrocytomas [1,95]. 

Moreover, CDKN2A/B is one of the criteria to diagnose High-grade astrocytoma with piloid features alongside a piloid cytology, frequent MAPK pathway gene alterations, loss of ATRX nuclear expression, and a distinct DNA methylation pattern [19].

## 8. 1p/19q Codeletion

Chromosomal deletion of both 1p and 19q, so called codeletion, represents an unbalanced translocation t(1;19)(q10;p10) [96,97] (Figure 1B). This alteration is associated with oligodendrogliomas and sensitivity to alkylating agent chemotherapy [98].

### 8.1. Classifying Marker

1p/19q is a key mutation that allows more accurate classification of tumors than histological evaluation [31]. Due to its prognostic significance, oligodendrogliomas with classic histological features remain a molecularly heterogeneous type that should be stratified according to 1p/19q status [54]. 1p/19q codeletion rates were 70.8% in oligodendroglioma grade 3 and 23.1% in astrocytoma grade 3 [99]. 1p/19q codeletion is closely linked to *IDH* mutations [38,100] and is nearly mutually exclusive with *ATRX* mutations [101]. According to the new classification, all oligodendrogliomas are 1p/19q codeleted [1].

### 8.2. Prognostic Factor 

1p/19q codeletion is a favorable prognostic factor (see Table 1) associated with a better PFS and OS [55,102,103] regardless of the detection method used [55,96,104]. The mutation indicates a relatively homogenous disease subtype [34,102]. Furthermore, isodeleted chromosome 1p predicted a favorable OS and PFS equivalent to codeleted 1p/19q, particularly in low-grade gliomas, but isodeletion of 19q only predicted prolonged PFS [55]. 

### 8.3. Predictive Factor

Loss of chromosome 1p/19q predicts both a persistent chemosensitivity and a favorable prognosis in LGGs that respond to TMZ [103]. Weller et al. reported that 1p/19q codeletion is a predictor of prolonged survival in patients receiving PCV (procarbazine, lomustine (CCNU), and vincristine) in addition to radiotherapy vs. radiotherapy alone [105]. Furthermore, PCV treatment is particularly effective in tumors with a high percentage of 1p/19q loss of heterozygosity and *IDH1* mutations [106,107].

## 9. Chromosome 7 Gain and Chromosome 10 Loss

Gliomas frequently have chromosomal alterations, however, chromosome 7 gain and chromosome 10 loss are of particular interest. There are nine possible combinations exhibiting both, gain of at least one arm on 7 and loss of at least one arm on 10. The most frequent constellation is complete chromosome 7 gain and complete chromosome 10 loss (79%), followed by 7+/10q−(5%), by 7p+/10−(5%), and by 7q+/10−(4%) [108].

### 9.1. Negative Prognostic Factor

7+/10− is a negative prognostic factor in gliomas. Particularly, in IDH-wildtype GBM, 7+/10− represents a hallmark molecular change. Astrocytic tumors not fulfilling the morphological criteria for GBM but carrying 7+/10− exhibit a clinical course similar to that of morphologically unequivocal GBM [108]. Therefore, in WHO CNS5, 7+/10− is one of the three genetic parameters to upgrade astrocytoma (grade 2 or 3), IDH-wildtype to glioblastoma, IDH-wildtype [1]. Three combinations, 7+/10−, 7q+/10−, and 7+/10q−, were linked to a poor prognosis, similar to that of GBM patients. As a result, all patients with 7+/10−, 7+/10q−, and 7q+/10− should be classified as having the prognostic 7+/10− signature [108].

Regardless of whether it is a complete or partial loss of chromosome 10, patients had significantly shorter survival compared to those with no chromosome 10 abnormalities [109]. Moreover, gain of whole chromosome 7 was associated with a 4.7-fold greater risk of tumor recurrence, even after correcting for surgical status and other genetic changes [110].

### 9.2. Positive Predictive Marker

*MGMT* is located on chromosome 10q26 and the loss of chromosome 10q is a form of *MGMT* inactivation. Richard et al. proved GBM patients with dual inactivation of *MGMT*, by hypermethylation of the *MGMT* promoter and by loss of the long arm of chromosome 10, have longer OS and PFS and receive greater benefit from TMZ treatment. Individuals with dual MGMT inactivation had a median OS of 21.5 months, compared to 12 months and 8.1 months, respectively, for groups with single MGMT inactivation by hypermethylation or 10q deletion. Furthermore, all long-term survivors (OS > 30 months) with a sustained response to TMZ treatment had dual MGMT inactivation [111].

## 10. MYB

*MYB* transcription factors are proto-oncogenes. Myb proto-oncogene like 1 (MYBL1) is a member of the *MYB* family. *MYB* alteration influences proliferation and differentiation of progenitor cells [112]. In gliomas, *MYB* gene alterations are detected more frequently in young children and typically affect the cerebral hemispheres [113]. cIMPACT-Now Update 4 reviewed the status of WHO grade 2 IDH-wildtype/H3-wildtype diffuse gliomas, including those with *MYB* or *MYBL1* rearrangements, and recommended the use of integrated diagnostics to link their histological and genetic characteristics [114]. Therefore, WHO CNS5 introduced a new type of glioma called diffuse astrocytoma, *MYB-* or *MYBL1*-altered belonging to the family of pediatric-type diffuse low-grade gliomas. Moreover, WHO CNS5 classifies diffuse astrocytomas, MYB- or MYBL1-altered as CNS WHO grade 1. Lastly, *MYB* gene is altered in nearly all Angiocentric gliomas [1].

### Positive Prognostic Factor 

In glioma patients with *MYB* and *MYBL1* mutations, the clinical course is generally indolent. Patients with these tumors have a prolonged disease course and good overall survival [114]. Chiang et al. reported that the 10-year OS is 90%, and 10-year PFS is 95%. Therefore, mutations in the *MYB* or *MYBL1* genes appear to be a positive predictor of prognosis [113].

## 11. MN1

Meningioma 1 (*MN1*) gene is a transcriptional co-regulator located on chromosome 22q [115,116]. Alterations in *MN1* frequently occur in astroblastoma, a type of gliomas. Astroblastoma is a neoplasm that primarily affects children, teenagers, and young adults, and it mainly involves the cerebral hemispheres. The term “astroblastoma” is thought to be misleading because these tumors are neither astrocytic nor blastic [117]. Therefore, WHO CNS5 has identified astroblastoma as “MN1-altered” to improve diagnostic clarity for this type. Even so, more research is needed to establish distinct histopathological and molecular characteristics that can identify MN1-mutated astroblastomas from morphologically identical neuroepithelial tumors with similar genetic changes [1].

### Positive Prognostic Factor

*MN1* is a positive prognostic factor [115,116]. Gliomas with upregulated *MN1* have better OS and PFS [116]. According to Lehman et al. *MN1* rearranged astroblastomas were associated with a favorable prognosis which was mainly in comparison to BRAF V600E-mutated pleomorphic xanthoastrocytoma [118].

## 12. MAPK Pathway 

The mitogen-activated protein kinase (MAPK) pathway comprises several key signaling components and phosphorylation events that play a role in tumorigenesis. These activated kinases transmit extracellular signals that regulate cell growth, differentiation, proliferation, apoptosis, and migration functions [119]. The MAPK pathway gene alterations most frequently affect *NF1*, followed by *BRAF* and *FGFR1* [120]. Alterations affecting genes encoding members of the MAPK pathway have previously been found to occur in up to 100% of CNS WHO grade 1 Pilocytic astrocytomas [121,122].

A tumor that has mutations in *FGFRs* and/or *BRAF* and morphologically resembles a diffuse glioma, according to the new WHO CNS5 classification, qualifies as diffuse low grade glioma, MAPK pathway-altered. On the other hand, a tumor with these mutations and neuroepithelial features can be classified as polymorphous low-grade neuroepithelial tumor of the young. Moreover, a KIAA1549–BRAF fusion is almost pathognomonic of pilocytic astrocytoma and high-grade astrocytoma with piloid features [114]. In both of these tumors *NF1* occurs frequently. Lastly, *BRAF* mutations are also found in pleomorphic xanthoastrocytoma [1].

### Positive Prognostic Factor 

The MAPK pathway activation is a predictor of a favorable patient outcome. Overall, patients with the MAPK pathway activation in the absence of *H3K27M* had a better prognosis (91% 5-year survival), whereas patients with *H3K27M* had a worse prognosis across all histological grades, suggesting that *H3K27M* is the dominant prognostic indicator [56]. A *KIAA1549:BRAF* fusion was associated with longer OS and PFS. This prognostic significance was regardless of the *FGFR1* status and the *FGFR1* immunohistochemical expression. On the other hand, tumors negative for a *KIAA1549:BRAF* fusion, the *FGFR1 pK656E* point mutation resulted in a significantly worse outcome, whereas the overexpression of *FGFR1* was related to a better prognosis [123].

## 13. EGFR

Epidermal growth factor receptor, also known as *HER1* or *ERBB1*, is a transmembrane receptor tyrosine kinase in the ERBB family [124]. *EGFR* overexpression and/or mutations play a central role in cell division, migration, adhesion, differentiation, and apoptosis [125]. Genetic alterations in *EGFR*—including mutations, rearrangements, alternative splicing, and focal amplifications—are the dominant receptor tyrosine kinase lesions in GBM. *EGFR* deletions and point mutations that keep *EGFR* in an active conformation are found in 57% of GBMs [126].

Among EGFR mutants, the most common is *EGFR*(△2–7), also called *EGFRvIII* [127,128,129]. *EGFR(*△2–7) is characterized by a 267 amino acid deletion in the extracellular domain, which results in a receptor that cannot bind ligand and is constitutively active [128]. *EGFR(*△2–7) is thought to represent a late event, occurring after *EGFR*-wildtype amplification. *EGFR* amplification and *EGFR*(△2–7) mutations might represent concerted evolutionary events that drive the aggressive nature of GBM by promoting invasion and angiogenesis via distinct signaling pathways [129]. 

### 13.1. Negative Prognostic Factor 

*EGFR* amplifications have been reported to indicate a much more aggressive tumor subpopulation [130]. Aibaidula et al. reported patients with EGFR-amplified LGGs had significantly shorter OS than those with EGFR non-amplified tumors (median OS 1.03 y vs. 2.67 y, *p* = 0.003) [66] (see Table 1). Labussière et al. suggested that the influence of *EGFR* status on prognosis could be more complicated; patients with *EGFR* amplifications had a better prognosis in the *TERT*-mutated context than patients with *TERT*-wildtype tumors. On the other hand, *EGFR*-wildtype GBM patients had longer survival with *TERT*-wildtype than patients with *EGFR*-wildtype and *TERT*-mutated [131]. Zou et al. showed that *EGFR* amplification and *IDH* mutations are mutually exclusive [38]. Therefore, further research is required to determine their multi-genic interaction. 

In LGG, *EGFR* mutations indicate increased lesion infiltration of specific immune cell types and a poor prognosis [132]. *EGFR*(△2–7) confers a growth advantage to GBM, and patients with *EGFR*(△2–7) mutations have significantly shorter survival. *EGFR*(△2–7) overexpression in the presence of *EGFR* amplification is the strongest indicator of a poor prognosis [133]. 

On top of that, in WHO CNS5 *EGFR* gene amplification is one of the criteria to upgrade IDH-wildtype diffuse astrocytic tumor in adults to glioblastoma, IDH-wildtype [1].

### 13.2. Promising Predictive Factor

Currently, intensive research is being done to develop drugs targeting EGFR. Monoclonal antibodies such as cetuximab and nimotuzumab have not been effective due to BBB (blood brain barrier) and their molecular weight. However, GC1118 is a novel anti-EGFR monoclonal antibody and has shown promising results. A phase II trial of GC1118 for recurrent GBM patients with EGFR amplification is underway (NCT03618667) [134]. Tyrosine kinase inhibitors (e.g., gefitinib and erlotinib) have failed to show remarkable improvement in patients with non-progressive or recurrent glioblastoma in various phase II clinical trials [135]. However, a new covalent-binding EGFR-TKI, CM93 showed better efficacy in pre-clinical than other EGFR-TKIs, which is promising [136]. Moreover, PI3K inhibitors, such as XL765, a dual mTOR and PI3K oral inhibitor, are currently examined in glioblastoma patients and a phase I trial is underway [135]. The recent study conducted by Zając et al. proved that inhibition of the PI3K/Akt/mTOR pathway sensitizes glioma cells to apoptosis upon temozolomide treatment [137]. Furthermore, epigenetic alterations also play a role in the development of the resistance to EGFR inhibitors. Treatment using epigenetic regulators, alone or in conjunction with EGFR inhibitors, offers a new hope for glioblastoma patients. For example, the combination of histone deacetylase (HDAC) inhibitor + EGFR inhibitor can prevent the development of the resistance in glioblastoma cells [138]. Finally, the development of hybrid compounds is another active area in glioblastoma therapeutics. For instance, Sahaquine contains hydroxamic acid and primaquine linked by dicarboxylic acid [139]. This hybrid molecule can selectively inhibit HDAC6 protein at nanomolar concentration. Moreover, Sahaquine suppresses P-glycoprotein activity, which contributes to glioblastoma drug resistance [140].

In summary, currently EGFR-targeting drugs do not prolong OS and PFS, but intensive research for new types of drugs is ongoing.

## 14. MGMT

O^6^-methylguanine-DNA methyltransferase is a DNA-repair protein that inhibits the cross-linking of double-stranded DNA by alkylating agents [141]. MGMT expression levels in gliomas may influence responses to alkylating agents. Promoter methylation regulates *MGMT* expression by epigenetic silencing of the *MGMT* gene [142]. The *MGMT* promoter is methylated in ~45% of glioma patients [143,144].

### 14.1. Positive Predictive Factor

*MGMT* promoter methylation has the most impact on clinical practice for patients with glioblastoma [145]. Patients with methylated *MGMT* benefit from TMZ, while patients without methylation do not [33,36,142,144,146,147,148,149], a phenomenon present in all age groups [59]. Moreover, double inactivation of *MGMT* by promoter methylation and loss of 10q result in greater sensitivity to TMZ than promoter methylation or absence of 10q alone [113]. A combination of both *IDH* mutations and *MGMT* promoter methylation was associated with the best response rates to TMZ [33,36]. Moreover, Roszkowski et al. found that patients with both *MGMT* promoter methylation and *IDH1* mutations receiving radiotherapy had a better prognosis than those with *MGMT* methylation alone [150]. However, Vuong et al. reported that not all GBM patients with methylated *MGMT* may benefit from TMZ, postulating that it is possible that only GBM patients mutated by *TERT* with *MGMT* methylation are sensitive [59].

### 14.2. Positive Prognostic Factor 

Not only *MGMT* is a positive predictive factor for TMZ therapy (see Table 1), but also *MGMT* is a positive prognostic marker. Many studies have reported that *MGMT* methylation predicts longer OS [33,81,151,152,153]. Zhang et al. conducted a meta-analysis of 15 studies reporting the effect of *MGMT* promoter methylation on OS by univariate analysis and 14 studies by multivariate analysis. The combined hazard ratios (HR) were 0.67 (95% CI 0.58–0.78) and 0.49 (95% CI 0.38–0.64), respectively. The pooled HR estimates for PFS were 0.72 (95% CI 0.55–0.95) by univariate analysis and 0.51 (95% CI 0.38–0.69) by multivariate analysis [143]. The survival time of glioblastoma patients with only an *IDH1* mutation was shorter than for patients with both *IDH1* mutations and *MGMT* methylation [40]. PFS was longer in patients with *MGMT* promoter methylation who received TMZ [33,36,57,154,155,156]. Furthermore, Shah et al. reported that methylation at different sites of the *MGMT* promoter results in various PFS [157].

However, there is evidence to suggest that *MGMT* is not a favorable prognostic factor in a selected group of patients. Boots-Sprenger et al. failed to identify a favorable prognostic association for *IDH1* mutations and *MGMT* promoter methylation in patients over 50 years of age [158]. Moreover, Nguyen et al. reported that *MGMT*-methylated patients showed improved survival only in the presence of *TERT* promoter mutations (*TERT-mt*) [159]. Finally, Dahlrot et al. discovered an association between *MGMT* methylation and OS starting at nine months following diagnosis but no association before that [160].

## 15. Mismatch Repair

Although the effectiveness of TMZ is largely dependent on the methylation status of the promoter of the MGMT gene, the integrity of the mismatch repair (MMR) system also plays a very important role. The MMR system is a protein complex including MSH2, MSH6, MLH1, and PMS2 proteins. MMR attempts to repair the O6-meG:T mismatch caused by TMZ by removing a patch of the newly synthesized strand containing thymine [161]. The MMR system recognizes mispaired O6-MeG:T and excises the newly synthesized strand, leaving the parental strand with O6-MeG intact. These futile cycles repeat, leading to cell cycle arrest and apoptosis. On the other hand, if the MMR system is defective then mutations accumulate leading to hypermutated phenotypes. Thus, the tumor is resistant to temozolomide, by not responding to TMZ-induced mispairing [162].

The predictive value of the MMR system is so important that Suwala et al. even proposed a new glioma type—primary mismatch repair-deficient IDH-mutant astrocytomas (PMMRDIA). PMMRDIA were histologically high-grade and were mainly found in younger patients (median age 14 years) and all of them had a defective MMR system. They also reported that compared to reference cohorts of other IDH-mutant gliomas, PMMRDIA had by far the worst clinical outcome with a median survival of only 15 months irrespective of histological or molecular features [163]. 

On the other hand, Caccese et al. in a multicenter study reported that MMR protein expression status did not affect survival in HGG patients. They also showed that alteration of MMR protein expression was statistically more frequent in grade 3 gliomas, in recurrent disease, in patients treated with temozolomide, and in IDH-mutant gliomas [164].

### 15.1. Hypermutation Phenotype 

The hypermutation phenotype is defined by a dramatic increase in the mutation rate. This phenomenon occurs rarely in newly-diagnosed gliomas, but common in recurrent tumors after the use of alkylating agents [165]. There are two main pathways to hypermutation. First, a de novo pathway associated with constitutional defects in DNA polymerase and MMR genes. Second, a more common post-treatment mechanism is related to acquired resistance in chemotherapy-sensitive gliomas that relapse following temozolomide treatment [166].

### 15.2. Questionable Predictive Value for Immune Checkpoint Inhibitors (ICI) 

Immune checkpoint inhibition is an attractive therapeutic avenue for hypermutated tumors. Pembrolizumab, a programmed cell death protein 1 (PD-1) inhibitor, was recently approved for the treatment of microsatellite instability–high, or MMR-deficient solid cancers in adults and children, for all tumor locations and histological types [167]. The most pronounced responses to ICI have been among tumors known to have high mutational burdens [168] such as subsets of non-small cell lung cancers [169], malignant melanomas [170] renal cell carcinoma [171], and MMR-deficient tumors [172]. However, in gliomas, the outcomes are less favorable. Touat et al. showed that MMR-deficient gliomas were characterized by a lack of substantial T-cell infiltrates, extensive intratumoral heterogeneity, and a low rate of response to ICIs. They stated that patients with hypermutated gliomas had worse median OS when treated with PD-1 inhibitors than patients treated with other systematic agents (16.10 months (95% CI 3.98–22.21) versus 8.07 (95% CI 2.79–15.08.21)) [166]. Moreover, the disappointing results of the Checkmate 143 trial (NCT02017717), which evaluated nivolumab and ipilimumab, has found no improvement in survival in patients with recurrent GBM [173]. Therefore, the usefulness of ICI in hypermutated glioma patients is questionable. 

## 16. Liquid Biopsies

**Figure 2:**
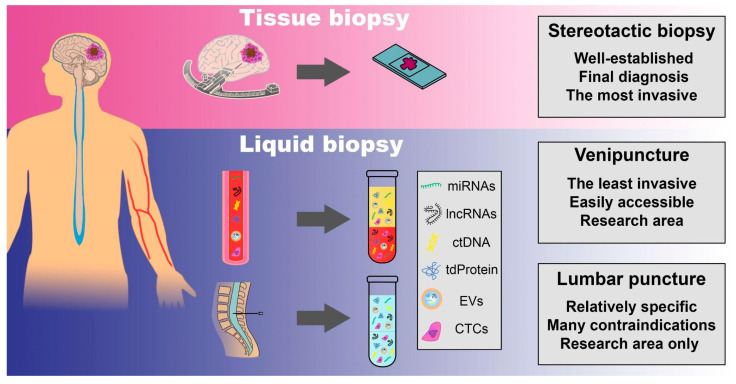
Obtaining the material to analyze biomarkers in glioma patients. The material can be obtained using tissue biopsy or liquid biopsies. A stereotactic biopsy is a well-established surgical procedure used to acquire tissue samples. Venipuncture and lumbar puncture are methods to obtain liquid biopsies. Circulating tumor cells (CTCs), extracellular vesicles (EVs), tumor-derived proteins (dtProteins), circulating tumor DNA (ctDNA), long non-coding RNAs (lncRNAs) and microRNA (miRNAs) are released into the bloodstream and cerebrospinal fluid (CSF) from primary tumors and metastatic deposits. Material collected by venipuncture, the least invasive technique, can be extracted, and plasma or serum can then be analyzed. The evaluation of circulating biomarkers in CSF is a relatively specific method, however it has many contraindications and currently remains only in the research area.

There has been a renaissance in molecular techniques over the last 20 years, including in real-time quantitative PCR (qPCR), FISH analysis, and next-generation sequencing (NGS) (Figure 1). On the other hand, small amount of material encourages for biological analyses from body fluids such as blood, cerebrospinal fluid (CSF), and urine, with CTCs, cell-free nucleic acids (cfNAs), ctDNA, and extracellular vesicles (EVs) all extracted for downstream analyses (Figure 2). This approach may also be beneficial for tracking dynamic changes in the tumor throughout therapy, given the minimally or non-invasive nature of the test. Moreover, most of these biomarkers have a short half-life (up to 3 h) so disintegrate quickly when present freely in plasma [174]. The multitude of available diagnostic options is at the stage of scientific research, which makes it difficult to compare methods. Furthermore, there is no widespread agreement among scientists regarding which nucleic acids (RNA or DNA), which biological fluids (serum, CSF, or urine), or which analytical technique (targeted or whole genome sequencing, PCR, or microarray) should be studied the most [175]. Therefore, it is hard to compare sensitivity and specificity of different methods. On top of that, not all patients provide their consent to particular diagnostic methods.

In gliomas, material to the analysis can be acquired primarily from blood or CSF. While blood drawing is a relatively simple procedure, a lumbar puncture (LP) has several contraindications. The most important is an intracranial space–occupying lesion with mass effect as well as a posterior fossa mass because it can lead to herniation of the cerebellar tonsils, regardless of the volume of CSF that is sampled [176]. Hence, patients should be carefully selected for LP.

Despite numerous limitations to overcome, liquid biopsies can be the future of personalized medicine due to their major advantages over tissue biopsies. Below we summarize various types of liquid biopsies. 

## 17. Circulating Tumor DNA 

Circulating tumor DNA (ctDNA) comprises small fragments of DNA (180–200 base pairs) released by tumor cells into the bloodstream, predominantly by cell death and apoptotic cells [177,178,179]. ctDNA has the potential to carry a wide spectrum of specific primary brain tumor mutations, and thus if detected in body fluids provides a valuable non-invasive or minimally invasive way to sample cancerous tissues (Figure 2) [174,180]. Moreover, the European Medicines Agency and the US Food and Drug Administration (FDA) have approved ctDNA tests for specific indications in the absence of evaluable tumor tissue [179].

Piccioni et al. found that half of primary brain tumor patients had detectable ctDNA [181], and Liang et al. proved that it is possible to distinguish a primary brain tumor from a metastasis based on ctDNA [182]. ctDNA concentrations vary depending on the cancer type, and GBMs produce extremely low plasma concentrations [183]. Nevertheless, ctDNA can be detected in body fluids such as CSF and blood, and the concordance rate between CSF ctDNA mutations and tumor DNA is quite high [184]. Li et al.’s analysis showed that the sensitivity of ctDNA detection increases when CSF is used instead of blood [185]. Compared to plasma ctDNAs, CSF ctDNAs more clearly represented the progressive mutational alterations of driver genes [185,186]. The existence of the blood-brain barrier (BBB) explains the limited concentration of ctDNA in glioma patients’ blood and the reduced sensitivity compared to CSF [174]. On the other hand, CSF cannot be collected easily and noninvasively for the diagnosis of glioma which limits the application of the CSF DNA analysis.

In a multivariable analysis, CSF ctDNA positivity remained a statistically relevant prognostic factor. CSF-positive subjects had a four-fold increased risk of death compared to CSF-negative subjects [64]. Bagley et al. reported that in patients with newly diagnosed GBM, high baseline plasma ctDNA concentrations were associated with worse OS and PFS regardless of other prognostic factors [62]. Moreover, Nørøxe et at. found that ctDNA levels changed during glioblastoma therapy, peaking before diagnostic surgery and declining as the cancer progressed [187].

However, there are still many challenges in detecting ctDNA which can limit the prognostic and predictive value. The most important is the detection method. CtDNA sequencing techniques must be extremely sensitive and specific to overcome the low concentration of ctDNA and the presence of DNA from normal cells, which can result in false positives. While qPCR is quick and inexpensive, it can only detect mutant allele fractions (MAF) greater than 10% [188]. Digital PCR (dPCR) tests using microfluidic platforms are highly sensitive and quantitative, and are widely used to measure ctDNA levels and it can detect MAF below 0.1% [189]. These methods are generally suited to investigating a small number of mutations and are often applied to analysis of cancer hot-spot mutations [179]. NGS-based approaches allow for high throughput analysis, can screen for undiscovered variants, and can also find structural variants and copy number variations, but they have a lower sensitivity (about 1%) than dPCR and higher cost [189]. Generally, there are three ways to increase the sensitivity and specificity, and consequently prognostic and predictive value of ctDNA. First, is to have a more precise machine. Second, is to increase the amount of collected blood. Lastly, we can search for many mutations in one genome molecule [190].

Another challenge is to reduce the background noise which can originate from white blood cells (WBC). Clonal hematopoiesis, a process that leads to expansion of mutations in peripheral blood cells, is an additional source of DNA that adds a layer of complexity when interpreting results. One way to overcome this problem is to sequence DNA profiles of WBC and compare them to analyzed ctDNA [191].

Because of the growing acceptance of ctDNA, the field is moving away from exploratory ctDNA studies and toward clinical trials, where ctDNA is used to influence decision-making [179].

## 18. MicroRNAs

MicroRNAs (miRNAs) are noncoding RNA molecules about 22 nucleotides long that influence gene expression by interacting with messenger RNAs (mRNAs) [192,193]. They are under sophisticated control. miRNAs are thought to influence up to 60% of protein-coding genes [193]. Many studies over the past few years have documented the control of miRNA metabolism and function by various mechanisms [194]. miRNAs can be assessed in body fluids such as blood, CSF, or urine as well as tissues, but the latter analysis may be hampered when only small amounts of neoplastic tissue are present: the assessment of somatic changes such as point mutations or 1p/19q codeletions requires an appropriate amount of material for genetic testing. Thus, peripheral blood miRNA expression is a potential source of material obtainable via a relatively non-invasive procedure (Figure 2) that could provide an innovative solution for assessing diagnosis, prognosis, and predicting responses to therapy with so-called “liquid biopsies” (see below).

### 18.1. Potential Classification Marker

Due to their participation in carcinogenesis and stability, miRNAs can serve as unique biomarkers for the minimally invasive diagnosis of glioblastoma [195,196]. Roth et al. investigated whether a specific blood-derived miRNA fingerprint could be defined in glioblastoma patients, and in doing so showed that miRNAs can be considered as biomarkers and their detection in the blood justifies the need for further testing [197]. In 2019, Wang et al. conducted a meta-analysis which showed that cell-free miRNA-21 is the most promising diagnostic miRNA for glioma detection, followed by miRNA-125 and miRNA-222 [198]. Moreover, ParvizHamidi et al. reported that both miRNA-21 and miRNA-26 were significantly upregulated in pre- and postoperative serum samples from glioblastoma patients [199]. In contrast, Wei et al. found that blood miRNA-125b levels were considerably lower in glioma patients than in the general population, with a clear downward trend in miRNA-125b levels as tumor stage increased [200]. Akers et al. reported that miRNAs from cisternal cerebrospinal fluid had sensitivity of 80% and specificity of 76% for glioblastoma detection whereas lumbar CSF had sensitivity of 28% and specificity of 95% [201]. According to Teplyuk et al., miRNA-200 family members are significantly elevated in the CSF of patients with brain metastases but not in individuals with any other pathological conditions, allowing differentiation between glioblastoma and metastatic brain tumors [202]. 

Nilsson et al. reported that platelets can absorb RNA-containing membrane vesicles in vitro and in vivo. Platelets from glioma patients absorbed vesicles containing mutant *EGFR*(△2–7), a well-known GBM biomarker (see above). Eighty percent of patients with *EGFR*(△2–7)-mutated GBM tumors also harbored the mutation in platelets compared to none in healthy controls. This conclusion is likely to apply to other tumor-related RNAs, as a glioma-associated signature was discovered after RNA profiling of glioma and healthy patients. Further research on upregulated RNAs could lead to the identification of new circulating biomarkers [203].

### 18.2. Potential Prognostic and Predictive Markers 

Over the past decade, there has been significant interest in the functional relevance of miRNAs as prognostic and predictive biomarkers. A number of meta-analyses have been conducted to investigate their prognostic significance. Upregulation of plasma miRNA-222, miRNA-155, miRNA-221, and miRNA-21 is associated with a worse prognosis [69,75]. Furthermore, there is a strong negative correlation between elevated miRNA-21 in serum and OS and PFS [71,73,195] (see Table 1). Glioblastoma patients with high levels of miRNA-10 family members in tissue had a much poorer survival rate than those with low levels of miRNA-10 [204]. 

In parallel, miRNA expression is often associated with responses to therapy, so miRNAs may also have a potential role as predictive biomarkers. Upregulated plasma levels of miRNA-223 and miRNA-125b-2 improved cell survival when treated with TMZ [205]. miRNA-125b-2 is overexpressed in GBM tissues and the corresponding stem cells (GBMSCs). Downregulation of miRNA-125b-2 expression in GBMSCs may allow TMZ to trigger apoptosis in these cells [206]. Moreover, Siegal et al. reported that miRNA-10b and miRNA-21 are predictive factors for bevacizumab (a vascular endothelial growth factor (VEGF) inhibitor) responses, discovering that the serum expression of these miRNAs was inversely linked to tumor size in patients receiving bevacizumab [207]. miRNA-181d could be a useful biomarker to determine which patients will benefit the most from TMZ therapy. Zhang et al. reported that *MGMT* expression inversely correlated with miRNA-181d expression in independent GBM samples [208]. While elevation of miRNA-181d may indicate a better response to TMZ, upregulation of miRNA-21 in a tumor sample, however, may indicate a poorer response [209]. Moreover, miRNA-21 expression is also closely associated with radio-resistance in diverse malignant glioma cell lines [210].

There have been many reports of miRNA up- or downregulation in glioma patients; however, definitive miRNA signatures for glioma classification or as prognostic or predictive biomarkers are still under evaluation. The patterns of miRNA expression in patients with gliomas are presented in Table 3.

## 19. Long Non-Coding RNA

Long non-coding RNAs (lncRNAs) are a new class of non-protein-coding transcripts that have been associated with cancer progression [225]. They comprise a wide variety of RNA transcripts with a length of more than 200 nucleotides but no significant protein-coding capacity [226]. One of the distinguishing characteristics of lncRNAs is their tissue- and cell type-specific expression pattern, which could be used to precisely categorize glioma subtypes and predict treatment responses [227] (Figure 2).

### 19.1. Prognostic Markers 

In 2020, Li et al. conducted a meta-analysis to evaluate the prognostic value of lncRNA expression in glioma patients. They showed a significant association between high lncRNA expression level and shorter OS (HR 2.09, 95% CI 1.68–2.58, *p* < 0.001). Moreover, lncRNA expression was significantly associated with a tumor diameter and malignancy [67]. Several other studies found that levels of lncRNAs are associated with prognosis. For example, LINC00152, LINC00319, and FAM225B were closely associated with unfavorable prognosis in glioma patients [228,229,230]. Moreover, Aslan et al. showed that lncRNA H19, AC091932.1, AC064875.1, AC010273.2, and AC131097.4 were negatively correlated with OS, whereas lncRNA FLG-AS1, AL138767.3, and ISX-AS1 were positively correlated with OS, indicating they play a protective role in LGG [231].

Recently, many studies have been published to find a set of lncRNAs with the best prognostic value. For this purpose, advanced statistical methods were applied to improve prognostic accuracy. For example, a set of six lncRNAs (AC005013.5, UBE2R2-AS1, ENTPD1-AS1, RP11-89C21.2, AC073115.6, and XLOC_004803) was an independent prognostic factor after adjusting for other clinical covariates. The set was able to stratify patients into high- and low-risk groups with significantly different survival (median 0.899 vs. 1.611 years, *p* = 3.87e−09, log-rank test) in the training cohort [232]. Moreover, Luan et al. identified 10 autophagy-associated lncRNAs with prognostic value *(PCBP1-AS1, TP53TG1, DHRS4-AS1, ZNF674-AS1, GABPB1-AS1, DDX11-AS1, SBF2-AS1, MIR4453HG, MAPKAPK5-AS1*, and *COX10-AS1)* in glioma patients using multivariate Cox regression analyses. The OS was shorter in the high-risk group than that in the low-risk group [HR = 5.307, 95% CI: 4.195–8.305; *p* < 0.0001] [233]. 

### 19.2. Predictive Markers

LncRNAs also have predictive value. It is proven that they affect chemotherapeutic drug resistance by regulating miRNA expression. For example, LINC00470 is reported to promote cell proliferation, invasion, and TMZ resistance through sponging miRNA-134 [234]. Furthermore, lncRNAs can interact with miRNA-21, which is related to processes regulating radio- and/or chemosensitivity [210] or miRNA-301a, which promotes radiation resistance. Thus, lncRNAs play important roles in the formation of tumor microenvironments and the acquisition of therapeutic resistance [235,236]. Recently, lncRNAs are receiving a large amount of attention as immunotherapy targets. Due to the fact that in gliomas the expression of immune-related lncRNAs is disrupted, and the clinical characteristics of glioma patients receiving immunotherapy are dependent on lncRNA expression [237]. 

## 20. Tumor-Derived Proteins 

Tumor-derived proteins may be detected in the bloodstream, making them suitable for noninvasive diagnostic verification (Figure 2). Several serum-based biomarkers have been studied in glioma patients to see whether they have substantial prognostic or predictive significance. So far, tumor derived proteins are one of the most extensively studied biomarkers in glioma patients [238].

### 20.1. Prognostic Markers

Iwamoto et al. found serum YKL-40 level was significantly lower in patients with no radiographic disease compared with patients with radiographic disease in both the grade 3 gliomas and the glioblastoma cohorts. In these patients, longitudinal increases in serum YKL-40 were linked to an increased risk of mortality. Moreover, increases in YKL-40 were linked to a lower survival rate in grade 3 gliomas (HR = 1.4, *p* = 0.0001) and glioblastomas (HR = 1.4, *p* < 0.0001). On the other hand, serum levels of YKL-40 in patients with LGG were not associated with radiographic disease status [68]. Hormigo et al. showed YKL-40 and MMP-9 can be monitored in patients’ serum and help confirm the absence of active disease in GBM and YKL-40 in grade 3 glioma patients. Additionally, they reported YKL-40 can be used as a predictor of survival in patients with HGG [239]. Vaitkiene et al., using the decision tree analysis, indicated that serums ANGPT1, TIMP1, IP10, and TGFβ1 are promising combinations of targets for glioma diagnosis. The serum protein profiles of ANGPT1, TIMP1, IP10, and TGFβ1 were linked with the presence of an astrocytoma irrespective of its malignancy grade, while OPN and IP10 were associated with GBM patient survival [240]. Furthermore, many angiogenic proteins are associated with survival in glioma patients. The plasma levels of IGFBP-2 and VEGF, the serum level of plasminogen activator inhibitor-1 (PAI-1) were inversely associated with PFS and OS [238].

### 20.2. Predictive Markers

So far, serum proteins have been predictive markers mainly for anti-angiogenic treatments. For example, Tabouret et al. indicated that high MMP2 plasma levels are related to longer survival in patients with recurrent HGG treated with bevacizumab but not with cytotoxic agents [241]. Moreover, Chinnaiyan et al. found IGFBP-5 as a possible predictive protein marker for combined treatment with bevacizumab, HDAC inhibitor vorinostat, and irinotecan in a limited study (*n* = 10 recurrent glioblastoma patients) [242].

## 21. Extracellular Vesicles 

Extracellular vesicles (EVs) are made up of an aqueous core containing soluble proteins and nucleic acids that are encased in a lipid bilayer [243]. EVs can be divided into two categories: in first, exosomes are released via exocytosis when multivesicular bodies fuse with the plasma membrane, and the second consists of microvesicles that shed directly from the cell membrane via budding [180]. Moreover, EVs can be classified according to their size, cargo, and density [244]. 

EVs are secreted by donor cells from the tumor niche and received by acceptor cells that may be located away from the tumor. EVs acquisition influences several signaling pathways through the internalization of various molecules (miRNAs, proteins, receptors, ligands, DNAs, and RNAs) (Figure 2) promoting processes such as invasion, angiogenesis, viability, migration, chemo-, and immunoresistance [244]. EVs are a reflection of the tumor’s extensive heterogeneity as well as its treatment adaptations [245]. Moreover, Gao et al. reported that the tumor microenvironment is altered by EV-mediated contact between glioma cells and non-glioma brain cells, which promotes tumor growth [246]. Recently, EVs emerged as a promising source of biomarkers for prognostic and predictive purposes. For example, EGFRvIII, an oncogenic glioma-specific growth factor receptor, has been shown to be present in EVs secreted by glioma cells [243,247]. Moreover, Wang et al. found that EGFR+ EVs can be used as glioma diagnostic and prognostic indicators. Using flow cytometry analysis, they demonstrated that EGFR expression in serum EVs can accurately distinguish high-grade glioma patients from low-grade glioma patients. Furthermore, EGFR in EVs correlates with the ki-67 labeling index in tumor tissue [175]. On the other hand, André-Grégoire et al. showed the TMZ treatment modulates glioma stem cells-released EVs. The release of extracellular vesicles was increased in temozolomide-resistant tumor cells. Moreover, TMZ increased the levels of cell adhesion proteins in extracellular vesicles [248]. Although EVs can be found in a variety of biofluids, it is still unclear which source is best for their isolation and prognostic value [249].

Similar to other liquid biopsy molecules, the isolation methods influence the sensitivity and specificity. It is especially observed in EVs where the density-based separation method is the most commonly used [188]. Density gradient centrifugation separates EVs and non-EV structures based on differences in buoyancy, making it currently the only isolation method that eliminates the majority of contaminants. However, the density gradient centrifugation is lacking a standardized protocol. EVs can be isolated through differential ultracentrifugation, rotor types, applied g-forces, and duration of centrifugation steps. Therefore, it results in different sensitivity and specificity, and consequently in various prognostic values [250]. 

## 22. Circulating Tumor Cells

Circulating tumor cells (CTCs) are cells that are released into the bloodstream from primary tumors and metastatic deposits [251] (Figure 2). In many recent studies, CTCs have been found in the blood of GBM patients [252,253,254,255,256,257,258,259,260,261]. GBMs discharge CTCs with invasive mesenchymal properties into the bloodstream, revealing mutations that were not observed in the primary tumor [256,260]. Sullivan et al. reported that acquired mutations in *EGFR, RB1*, and *SETD2* were absent in the primary tumor but were present at all metastatic sites [260]. Liu et al. reported that GBM-derived CTCs possess a cancer stem cell-like phenotype with resistance to radiation, chemotherapy, and stress-induced apoptosis [255]. Moreover, CTCs are valuable for tumor characterization in cases where tissue biopsies are difficult to obtain or the acquired tissue is of poor quality [255]. 

CTCs are present at as few as one cell per 10^9^ in the blood of patients with metastatic cancer. Detecting CTCs with high specificity and sensitivity is technically challenging [262]. The isolation methodologies of CTCs can have an impact on quality and quantity of the specimen, and consequently on sensitivity and specificity. Therefore, choosing the isolation method and source of CTCs can influence their prognostic and predictive value. There are several methods to isolate CTCs from the bloodstream. Immunomagnetic positive enrichment, immunomagnetic negative enrichment, microfluidic positive enrichment, size selection methods, and density centrifugation are the most popular. In the immunomagnetic positive enrichment, specific antibodies with ferritic properties attach to CTCs which are then separated from the blood using magnetic force. In immunomagnetic negative enrichment, ferritic antibodies target leukocytes, primarily using CD-45, and then they are wash out living CTCs, which in theory, should be the only nucleated cells. 

CellSearch^®^ (Menarini Silicon Biosystems Inc., Huntington Valley, PA, USA) is the only FDA-approved platform for CTCs isolation. It uses a combination of positive and negative immunomagnetic enrichment [263]. CellSearch is a validated method for CTCs enumeration in breast cancer [264], colon cancer [265], and prostate cancer [266]. However, CellSearch cannot be used for non-epithelial tumors, as it only targets EpCAM. Therefore, its usefulness in gliomas is questionable. Moreover, CellSearch does not provide opportunities to develop custom assays and CTCs can have different antigens, for example EGFR. As a result, this technology could not detect all CTCs and have reduced sensitivity or false negative results. 

Due to the lack of regularly expressed tumor markers and high inter- and intra-tumor heterogeneity, detecting glioma CTCs has been difficult [252]. Despite challenges in isolating, detecting, and diagnosing CTCs, a number of studies have now been published that outline new and effective detection strategies, but none has yet received widespread recognition and validation that would progress clinical acceptance [257,259,267]. The presence of CTCs correlates with the risk of metastasis and the frequency of relapse after surgery [253].

CTCs can serve as a stratification of patients with metastatic disease. In metastatic breast cancer the prognostic efficacy of CTCs has been proven. No study has yet been conducted in glioblastomas to assess how the presence and level of CTCs in the blood would influence survival in metastatic disease.

CTCs are promising prognostic and predictive factors; however, they are in the area of research and more studies are required to implement this novel technology in clinical practice.

## 23. Conclusions

Many biomarkers have been reported and are now clinically used in the management of neuro-oncology patients. They now play a crucial role in improving diagnostic accuracy, determining prognosis, and predicting treatment responses. Moreover, the 2021 WHO CNS classification put the highest emphasis on molecular markers than ever before.

### 23.1. Prognostic Markers

In adult patients, *IDH* mutations have the greatest prognostic significance, and a number of robust meta-analyses have shown that *IDH* mutations are associated with longer OS and PFS. The most favorable clinical outcomes are in patients with a combination of *IDH* mutation and 1p/19q codeletion. On the other hand, *CDKN2A* mutation indicates the highest malignancy grade in the group of diffuse, IDH-mutant astrocytomas. Additionally, *TERT* promoter mutations, *EGFR* alterations, and 7+/10− upgrade IDH-wildtype astrocytomas to glioblastomas. In pediatric patients, *H3F3A* alterations are the most important markers which predict the worse outcome. *MYB, MN1,* and MAPK pathway alterations, however, are positive prognostic factors. 

### 23.2. Predictive Markers

*MGMT* promoter methylation has the greatest clinical significance in predicting responses to TMZ. 1p/19q codeletion and loss of chromosome 10 are also positive predictive markers for the TMZ response. On the other hand, MMR defects lead to hypermutation phenotype and predict poor response to TMZ. Surprisingly, gliomas with hypermutation phenotype, have shown no improvement in outcomes after immune checkpoint inhibitors treatment. So far, EGFR alterations are promising predictive factors for novel targeted therapies and more clinical trials are required.

### 23.3. Liquid Biopsies 

miRNAs, lncRNA, ctDNA, extracellular vesicles, tumor-derived proteins, and CTCs are promising diagnostic, prognostic, and predictive biomarkers, but further work is needed to implement this novel technology in clinical practice. There is a need for minimally invasive methods to detect genetic biomarkers for the molecular characterization of brain tumors. A complete and comprehensive understanding of the genomic alterations that trigger gliomas continues to be essential for diagnostics, prognostics, and targeted therapies.

## Figures and Tables

**Table 1:** Families and types of gliomas according to the 2021 World Health Organization Classification of Tumors of the Central Nervous System [1].

Families	Types
Adult-type diffuse gliomas	Astrocytoma, IDH-mutant
	Oligodendroglioma, IDH-mutant, and 1p/19q-codeleted
	Glioblastoma, IDH-wildtype
Pediatric-type diffuse low-grade gliomas	Diffuse astrocytoma, *MYB*- or *MYBL1*-altered
	Angiocentric glioma
	Polymorphous low-grade neuroepithelial tumor of the young
	Diffuse low-grade glioma, MAPK pathway-altered
Pediatric-type diffuse high-grade gliomas	Diffuse midline glioma, H3 K27-altered
	Diffuse hemispheric glioma, H3 G34-mutant
	Diffuse pediatric-type high-grade glioma, H3-wildtype and IDH-wildtype
	Infant-type hemispheric glioma
Circumscribed astrocytic gliomas	Pilocytic astrocytoma
	High-grade astrocytoma with piloid features
	Pleomorphic xanthoastrocytoma
	Subependymal giant cell astrocytoma
	Chordoid glioma
	Astroblastoma, *MN1*-altered

**Table 2:** Prognostic biomarkers in gliomas.

Biomarker	Prognostic Value	OS (HR) 95% CI	PFS (HR) 95% CI	*n*	CNS WHO Grade	Reference
*IDH* mutation	Positive	0.241 (0.107–0.544)	NA	98	4	[40]
*IDH* mutation	Positive	0.33 (0.25–0.42)	0.38 (0.21–0.68)	2190	AGG	[38]
*IDH* mutation	Positive	0.20 (0.06–0.58)	0.14 (0.05–0.38)	108	LGG	[34]
1p/19q codeletion	Positive	0.33	0.34	79	4	[33]
1p/19q codeletion	Positive	0.30 (0.12–0.75)	0.34 (0.17–0.69)	203	2	[54]
1p/19q codeletion	Positive	0.43 (0.35–0.53)	0.63 (0.52–0.76)	3408	AGG	[55]
MAPK pathway alterations	Positive	0.19 (0.07–0.50)	NA	64	AGG	[56]
*ATRX*	Positive	0.36 (0.17–0.81)	NA	1206	AGG	[53]
m*MGMT* (TMZ-treated PT)	Positive	0.59 (0.37–0.94)	NA	274	4	[36]
m*MGMT*	Positive	0.42 (0.38–0.45)	0.43 (0.38–0.48)	5103	4	[57]
m*MGMT* (TMZ-treated PT)	Positive	0.46 (0.41–0.52)	0.48 (0.40–0.57)	7888	4	[58]
m*MGMT* (TMZ-free)	No effect	0.97 (0.91–1.03)	0.76 (0.57–1.02)	7888	4	[58]
*TERT*-mut (m*MGMT* PT)	Positive	0.73 (0.55–0.98)	NA	2819	AGG	[59]
*TERT*-mut (*MGMT*-free)	Negative	1.86 (1.54–2.26)	NA	2819	AGG	[59]
*TERT*-mut	Negative	1.37 (1.08–1.76)	1.37 (1.08–1.72)	785	AGG	[60]
*TERT*-mut	Negative	1.38 (1.15–1.67)	1.31 (1.06–1.63)	11519	LGG	[61]
High cfDNA	Negative	1.82 (0.61–5.42)	NA	42	4	[62]
High ctDNA	Negative	2.43 (1.19–4.95)	2.19 (1.26–3.81)	62	4	[63]
High ctDNA	Negative	NA	NA	85	AGG	[64]
*H3F3A*	Negative	4.27 (1.3–14.5)	NA	42	4	[45]
*CDKN2A*	Negative	2.2	2.1	2193	AGG	[65]
*EGFR* amplification	Negative	0.43 (0.24–0.77)	NA	718	LGG	[66]
lncRNA	Negative	2.09 (1.68–2.58)	NA	1415	AGG	[67]
Elevated serum YKL-40	Negative	1.4 (1.2–2.0)	NA	343	HGG	[68]
miRNA-221 upreg.	Negative	1.66 (1.34–2.04)	1.14 (1.02–1.26)	1069	AGG	[69]
miRNA-221 upreg.	Negative	2.13 (1.05–4.31)	NA	50	4	[70]
miRNA-221 upreg.	Negative	1.269 (1.054–1.527)	NA	4708	AGG	[71]
miRNA-155 upreg.	Negative	1.4 (1.19–1.63)	NA	1259	AGG	[72]
miRNA-21 upreg.	Negative	1.91 (1.34–2.73)	1.23 (0.41–3.72)	1059	AGG	[73]
miRNA-21 upreg.	Negative	1.681 (1.265–2.097)	NA	1681	AGG	[74]
miRNA-21 upreg.	Negative	1.591 (1.278–1.981)	NA	4708	AGG	[71]
miRNA-222 upreg.	Negative	2.09 (1.00–4.37)	NA	50	4	[70]
miRNA-222 upreg.	Negative	1.72 (1.31–2.26)	1.02 (0.86–1.22)	1546	AGG	[75]
miRNA-15b upreg.	Negative	1.584 (1.199–2.092)	NA	4708	AGG	[71]
miRNA-148a upreg.	Negative	1.122 (1.023–1.231)	NA	4708	AGG	[71]
miRNA-196 upreg.	Negative	1.877 (1.033–3.411)	NA	4708	AGG	[71]
miRNA-210 upreg.	Negative	1.251 (1.010–1.550)	NA	4708	AGG	[71]

Abbreviations: AGG—all grade gliomas; *ATRX*—a-thalassemia/mental retardation X-linked syndrome; *CDKN2A*—Cyclin-dependent kinase inhibitor 2A; ctDNA—circulating tumor DNA; *EGFR*—epidermal growth factor receptor; HR—hazard ratio; *IDH*—isocitrate dehydrogenase; LGG—lower-grade glioma; *mMGMT*—MGMT methylated; *MGMT*—O 6-methylguanine-DNA methyltransferase; miRNA—microRNA; *n*—number of patients; NA—non-accessible; OS—overall survival; PFS—progression-free survival; PT—patients; *TERT*—human telomerase reverse transcriptase; *TERT*-mut—TERT promoter mutated glioma; TMZ—temozolomide; upreg.—upregulated; CNS WHO grade—Central Nervous System World Health Organization Grade; lncRNA—long non-coding RNA.

**Table 3:** Upregulated and downregulated miRNAs in gliomas.

miRNAs Upregulated in Gliomas			miRNAs Downregulated in Gliomas		
miRNA	Sample	References	miRNA	Sample	References
miRNA-21	tissue, blood, CSF	[71,74,199,211,212,213,214,215,216,217,218,219]	miRNA-137	tissue	[216,219,220]
miRNA-221	tissue, blood	[71,213,214,221,222]	miRNA-342-3p	tissue, blood	[197,219]
miRNA-10	tissue, blood, CSF	[204,216,220,223]	miRNA-124	tissue	[71,217]
miRNA-222	tissue, blood, CSF	[70,75,198]	miRNA-181a, miRNA-181b, and miRNA-181c	tissue	[221]
miRNA-210	tissue, blood	[71,220]	miRNA-31	tissue	[216]
miRNA-17	tissue	[214]	miRNA-101	tissue	[216]
miRNA-155	tissue	[72,215]	miRNA-222	tissue	[216]
miRNA-576-5p	blood	[224]	miRNA-330	tissue	[216]
miRNA-340	blood	[224]	miRNA-7	tissue	[216]
miRNA-626	blood	[224]	miRNA-7-5P	blood	[224]
miRNA-630	tissue	[215]	let-7g-5p	blood	[224]
miRNA-1260	tissue	[215]	miRNA-320	blood	[224]
miRNA-542-5p	tissue	[215]	miRNA-125b	blood	[200]
miRNA-142-5p	tissue	[215]	miRNA-29	blood	[220]
miRNA-106a-5p	blood	[220]	miRNA-197	blood	[220]
miRNA-185	blood	[220]	miRNA-205	blood	[220]
miRNA-125	CSF, blood	[198]	miRNA-485	blood	[220]
miRNA-15b	tissue	[71]	miRNA-106	tissue	[71]
miRNA-148a	tissue	[71]			
miRNA-196	tissue	[71]			
miRNA-15a	tissue	[217]			
miRNA-16	tissue	[217]			
miRNA-23a	tissue	[217]			
miRNA-9	tissue	[217]			
